# The Missing Patient After Childbirth: A Health-System Framework for Postpartum Continuity of Care in Asia

**DOI:** 10.3390/healthcare14213640

**Published:** 2026-10-27

**Authors:** Nam Hoang Tran, Dolgorsuren Aldartsogt, Ngoc Bao Dang, Chung Thi Kim Le, Kanchana Piboon

**Affiliations:** 1 International Center, Tokushima University, Tokushima 770-8502, Japan; tran@tokushima-u.ac.jp; 2 School of Biomedicine, Mongolian National University of Medical Sciences, Ulaanbaatar 14210, Mongolia; dolgorsuren.a@mnums.edu.mn; 3 Internal Medicine Ward, Sun Group International Hospital, Hanoi 10000, Vietnam; bndang.nursing.research@gmail.com; 4 Institute for Preventive Medicine and Public Health, Hanoi Medical University, Hanoi 10000, Vietnam; lekimchung@hmu.edu.vn; 5 Faculty of Nursing, Burapha University, Chonburi 20131, Thailand

**Keywords:** postpartum health, postnatal care, maternal health, women’s health, Sustainable Development Goals, universal health coverage, midwifery, nursing, continuity of care, Asia, life-course health

## Abstract

Maternal survival remains an urgent global-health priority, yet survival does not necessarily imply recovery, wellbeing, or long-term health. After childbirth, women may experience persistent physical, psychological, reproductive, cardiometabolic, nutritional, and social challenges that extend beyond conventional maternity care. Although contemporary postnatal guidance increasingly recognizes maternal wellbeing and continuity, an important operational gap remains between time-limited maternity services and longitudinal women’s healthcare. This narrative review synthesizes peer-reviewed literature, international guidance, and selected national policy evidence to examine the health-system gap between time-limited maternity services and longitudinal women’s healthcare and proposes a Postpartum Women’s Health Continuum (PWHC) to address this transition. Japan, Sri Lanka, Thailand, and Mongolia are used as purposively selected health-system exemplars to illustrate institutionalized postpartum support, community-based continuity, integration within universal health coverage, and adaptation to geographic constraint. We propose a PWHC encompassing six interconnected domains and emphasizing universal early continuity, risk-stratified follow-up, nurse- and midwife-enabled coordination, selective use of digital health, and explicit handover of unresolved or pregnancy-associated risks into continuing care. As 2030 approaches, maternal-health success should be judged not only by survival, but also by recovery and long-term health.

## 1. Introduction

Reducing preventable maternal mortality remains one of the most important commitments of global health. The Sustainable Development Goals (SDGs) call for the global maternal mortality ratio to fall below 70 deaths per 100,000 live births by 2030, while the broader SDG framework emphasizes health, gender equality, social inclusion, and economic participation across the life course [1,2]. Despite substantial improvements over recent decades, recent estimates indicate that approximately 260,000 women died during or following pregnancy and childbirth in 2023, demonstrating that SDG Target 3.1 remains far from achieved [1]. Maternal survival must therefore remain a central policy and clinical priority. Survival, however, represents only one dimension of maternal health. A woman may survive pregnancy and childbirth while experiencing persistent pain, anemia, urinary or fecal incontinence, pelvic-floor dysfunction, depression, anxiety, breastfeeding difficulties, sexual dysfunction, unmet contraceptive needs, persistent hypertension, impaired glucose metabolism, sleep deprivation, social isolation, or substantial disruption of work and family life. Some problems become evident immediately after childbirth, whereas others persist or emerge months later.

Contemporary international guidance has already moved beyond a narrow mortality-based understanding of maternal health. The World Health Organization (WHO) defines a positive postnatal experience in terms of health, wellbeing, information, reassurance, support, and respectful care and explicitly recognizes postnatal care as important for both short- and long-term outcomes [3]. Wojcieszek et al., in describing the development of these WHO recommendations, similarly emphasize strengthening the continuum of maternal and newborn care rather than treating postnatal services as a single, isolated event [4]. Comparative examination of major clinical guidelines also shows broad agreement that postpartum care should address multiple aspects of maternal recovery and wellbeing, although the timing, scope, and organization of follow-up differ across systems [5]. The problem addressed in this narrative review is therefore not that global health has conceptually ignored postpartum women. Rather, we argue that the major remaining problem is operational continuity.

Pregnancy commonly brings women into frequent contact with health services. Clinical surveillance intensifies around childbirth. After delivery, however, the organization of care changes rapidly. Attention appropriately expands toward infant feeding, vaccination, growth, and development. Maternal care frequently remains concentrated in the early postnatal period, while persistent psychological, cardiometabolic, pelvic-floor, reproductive, or social problems may require services outside maternity care. Obstetric teams may consider the pregnancy episode concluded before primary-care, rehabilitation, mental-health, or chronic-disease services have clearly assumed responsibility. The resulting discontinuity has previously been described through the concept of the ‘fourth trimester.’ Tully et al. argued that the months after childbirth constitute a critical transition characterized by substantial unmet maternal-health needs [6]. The American College of Obstetricians and Gynecologists (ACOG) subsequently emphasized that postpartum care should be regarded as an ongoing process rather than a single encounter [7]. More recent systematic evidence indicates that alternative delivery strategies, including earlier contacts, multidisciplinary approaches, home-based services, and telehealth, may improve elements of postpartum care [8]. Nevertheless, qualitative and implementation studies continue to show that women, particularly after complicated pregnancies, can experience fragmented pathways and uncertainty regarding where responsibility for their ongoing health lies [9,10,11]. We describe this woman as the ‘missing patient after childbirth.’ The term does not imply that postpartum women literally receive no healthcare, nor is it intended as a synonym for low service utilization or non-attendance. Rather, it describes a health-system discontinuity in which the woman ceases to be managed as an integrated maternity patient before responsibility for unresolved postpartum needs or pregnancy-associated risks has been clearly assumed by a longitudinal source of care.

This paper argues that closing this transition represents an unfinished component of the SDG agenda. It further proposes a Postpartum Women’s Health Continuum (PWHC) connecting maternity care to longitudinal health through six interrelated domains. The framework was developed through a narrative synthesis of peer-reviewed research, international guidance, and selected national policy and health-system evidence, as described below. To illustrate how this principle can be implemented under contrasting conditions, we draw on Japan, Sri Lanka, Thailand, and Mongolia. These countries were selected purposively as analytical exemplars rather than as statistically representative cases. They should not be interpreted as representing postpartum healthcare across Asia, which is highly heterogeneous in healthcare financing, workforce capacity, geography, service organization, cultural practices, and maternal-health infrastructure. Selection considered the presence of identifiable postpartum-continuity mechanisms, variation in health-system organization and service-delivery context, availability of sufficiently documented evidence, and analytical complementarity across cases. During the narrative synthesis, four recurring health-system functions became particularly salient: institutionalized postpartum support, community-based continuity, integration within universal health coverage, and adaptation to geographic distance. Japan, Sri Lanka, Thailand, and Mongolia were retained as illustrative cases of these respective functions.

## 2. Methodology

### 2.1. Literature Identification and Narrative Synthesis

This article was developed as a narrative and conceptually oriented review rather than a systematic review or meta-analysis. The literature was identified through iterative searches of major biomedical and multidisciplinary databases, including PubMed and Google Scholar, together with targeted searches of authoritative international and national sources, including the WHO, United Nations agencies, the International Labor Organization (ILO), and relevant national health and government agencies. Searches were conducted during March–August 2026, with additional targeted searches undertaken during manuscript development and revision.

Search concepts included combinations of terms relating to postpartum or postnatal care, continuity of care, maternal morbidity, women’s health, primary care, mental health, pelvic-floor recovery, hypertensive disorders of pregnancy, gestational diabetes, sexual and reproductive health, breastfeeding, social and occupational wellbeing, nursing, midwifery, telemedicine, universal health coverage, and health-system transitions. Country-specific searches were additionally conducted for Japan, Sri Lanka, Thailand, and Mongolia. Country selection was purposive and iterative rather than statistically representative. Candidate country examples were considered according to four criteria: the presence of identifiable mechanisms supporting postpartum continuity of care; variation in health-system organization and service-delivery context; availability of sufficient policy, programme, or empirical documentation; and the ability of the case to contribute a distinct analytical perspective to the framework. The four countries ultimately retained were selected because they provided contrasting examples of postpartum continuity under different system conditions. The organizing functions highlighted in the comparative synthesis were refined during the narrative review process rather than imposed as rigid a priori selection categories. Through iterative comparison, Japan was interpreted as illustrating institutionalized postpartum support, Sri Lanka community-based continuity, Thailand integration within universal health coverage, and Mongolia adaptation to geographic distance. These functions were therefore used as analytical lenses for synthesis, not as predetermined inclusion criteria.

Evidence was selected purposively according to its relevance to the central question of how women transition from time-limited maternity care to continuing health services after childbirth. Priority was given to recent systematic reviews, major clinical or policy guidance, implementation studies, and authoritative national or international documents. Seminal earlier publications were retained where they provided important conceptual or historical foundations. The literature was synthesized narratively across recurring domains of postpartum health and health-system continuity. The country examples were selected as analytical exemplars illustrating contrasting system functions, institutionalized postpartum support, community-based continuity, integration within universal health coverage, and adaptation to geographic distance, rather than as a representative sample of Asian health systems.

### 2.2. Development of the Postpartum Women’s Health Continuum

The PWHC was developed through an iterative narrative synthesis rather than through a formal consensus methodology. During review of the literature, recurring maternal-health needs extending beyond the immediate postnatal period were grouped into broader conceptual categories. These categories were refined into six interconnected domains: physical recovery and function, mental health, cardiometabolic health, sexual and reproductive health, breastfeeding and nutrition, and social and occupational wellbeing. The domains were intended to capture clinically and socially important areas in which postpartum needs may persist beyond conventional maternity-care timelines, rather than to reproduce a single existing postpartum-care framework.

A second level of synthesis focuses on the health-system mechanisms required to maintain continuity across these domains. Evidence concerning postnatal contact, primary-care transition, risk-based follow-up, fragmented referral pathways, and longer-term management of pregnancy-associated conditions was compared with relevant international guidance. From this synthesis, three organizing principles were formulated: universal early continuity, risk-stratified follow-up, and explicit handover into continuing care. Existing WHO recommendations and other postpartum-care guidance informed these principles, but the PWHC as a whole was not directly adopted from any single pre-existing framework. The resulting structure was subsequently reviewed and refined among the authors and considered against the four country exemplars to assess its conceptual coherence across differing health-system contexts. This process represented author-based conceptual refinement rather than a formal expert-consensus exercise.

## 3. The Postpartum Woman Between Health-System Boundaries

The historical organization of maternal healthcare reflects the clinical urgency of pregnancy and childbirth. Hemorrhage, hypertensive emergencies, sepsis, thromboembolic disease, and other obstetric complications can deteriorate rapidly. Systems designed around antenatal risk identification, skilled birth attendance, emergency obstetric care, and immediate postpartum monitoring have therefore prevented countless maternal deaths. Many forms of postpartum morbidity, however, do not fit neatly within these acute-care timelines. A woman may be medically stable at discharge but continue to experience substantial limitations in physical function, psychological wellbeing, continence, sexual health, or metabolic recovery. Evidence concerning women’s uptake of routine postnatal services shows that attendance is shaped not only by service availability but also by perceived need, cultural expectations, financial and transport constraints, competing childcare responsibilities, quality of care, and relationships with healthcare professionals [12]. These findings are important because they challenge the common assumption that failure to attend postpartum care is predominantly a matter of individual motivation, or a matter of means and accessibility in some circumstances.

The organization of care itself matters. Cameron et al. demonstrated that enhanced postpartum transition programmes can improve engagement with primary care, illustrating how active handover may reduce the gap between obstetric services and longer-term healthcare [9]. In a randomized clinical trial, Clapp et al. showed that default scheduling combined with tailored messaging increased postpartum primary-care engagement, again suggesting that continuity can be improved by changing health-system design rather than simply instructing women to seek follow-up [10]. Qualitative research among women after high-risk pregnancy similarly demonstrates the confusion that may arise when patients must navigate multiple specialties and determine for themselves who is responsible for ongoing health concerns [11]. These findings point toward a fundamental distinction. Healthcare systems may successfully complete an obstetric episode without successfully completing a health transition. The latter requires unresolved problems to be recognized, assigned to an appropriate professional or service, followed until stabilized, and explicitly transferred when continuing care is needed. A referral letter by itself does not constitute continuity if no mechanism confirms that the woman has entered the next stage of care.

## 4. Postpartum Health and the Unfinished SDG Agenda

The SDGs provide a useful framework because postpartum women’s health intersects with several goals that are often treated separately in policy and clinical practice. This cross-cutting relevance reinforces the need to view postpartum health not as a narrow extension of obstetric care, but as a life-course issue linking clinical care, prevention, gender equality, social protection, and economic participation. Maternal mortality under SDG 3.1 is the most obvious connection, but postpartum care also relates directly to noncommunicable disease and mental health under Target 3.4, sexual and reproductive healthcare under Target 3.7, UHC under Target 3.8, unpaid care and reproductive rights under SDG 5, decent work under SDG 8, and social inclusion under SDG 10 [2]. The goal is therefore not to shift attention away from maternal survival, but to broaden the definition of maternal-health success from survival alone to survival accompanied by recovery, wellbeing, and long-term health.

WHO guidance on integrating sexual and reproductive health into primary healthcare reinforces this broader interpretation of UHC [13]. Many postpartum needs do not require repeated specialist obstetric care and could be managed or coordinated within primary healthcare if transitions were better designed. WHO’s recent guidance on networks of care similarly emphasizes linked systems in which referral and communication connect different levels and providers rather than leaving patients to navigate fragmented services independently [14]. This principle is also relevant to the evolving role of nurses and midwives. WHO’s guidance on transitioning toward midwifery models of care emphasizes continuity, person-centeredness, and appropriate professional roles throughout reproductive healthcare [15]. A life-course postpartum model therefore does not necessarily require expansion of specialist physician encounters. It requires clarity about which professional delivers which function and how escalation occurs when additional expertise is necessary. The SDG framework also extends the discussion beyond clinical medicine. Unpaid care and domestic work are explicitly recognized under SDG 5.4, while employment and decent work are central to SDG 8. The International Labor Organization (ILO) has documented persistent global gaps in maternity protection, parental leave, breastfeeding support, childcare, and other care-related policies [16]. These structural conditions matter clinically. Advice to sleep adequately, attend multiple appointments, maintain breastfeeding, exercise, and monitor chronic disease may be unrealistic for a woman who has little childcare support, insecure employment, substantial unpaid care responsibilities, or long travel requirements. Postpartum health should therefore be understood not simply as an obstetric issue but as a point where healthcare, gender, employment, and social policy intersect.

### 4.1. Physical Recovery and Function

A comprehensive postpartum pathway should begin with physical recovery but should not define recovery exclusively as absence of acute complication. The clinically meaningful question is whether a woman is regaining function. Pelvic-floor morbidity illustrates this distinction clearly. Nestor et al. found that bothersome pelvic-floor dysfunction can persist into the postpartum period and meaningfully affect quality of life [17]. Such problems may include urinary or fecal incontinence, prolapse symptoms, pain, and sexual dysfunction. Even when these symptoms do not require urgent care, they may affect exercise, social confidence, relationships, work participation, and willingness to engage in future pregnancy. An additional challenge is that women may perceive persistent pain, incontinence, fatigue, psychological distress, or other postpartum symptoms as normal consequences of childbirth and therefore may not recognize a need to seek professional care [18,19]. Postpartum systems should consequently combine accessible care pathways with proactive assessment and clear guidance about which symptoms warrant further evaluation.

There is also growing evidence that targeted interventions may improve recovery. Beamish et al.’s systematic review and meta-analysis found that postpartum exercise interventions can influence pelvic-floor outcomes and diastasis recti abdominis [20]. More recent meta-analytic evidence indicates that structured training interventions can improve postpartum urinary incontinence [21]. These studies illustrate why persistent postpartum symptoms should not simply be normalized as inevitable consequences of childbirth. The functional perspective also helps prevent overmedicalization. Not every woman requires specialist rehabilitation. Some need reassurance and basic advice, while others need physiotherapy or specialist assessment. The purpose of a longitudinal system is to differentiate between expected recovery and morbidity requiring intervention.

Although physical recovery and function constitute one of the six PWHC domains, functional recovery is not intended to be restricted to physical morbidity alone. Overall postpartum function may reflect interacting influences from several domains, including pain or pelvic-floor dysfunction, mental-health symptoms, breastfeeding-related difficulties, sleep disruption, caregiving demands, social circumstances, and return to occupational or community roles [17,20,21,22,23,24]. Functional status may therefore serve as a cross-cutting outcome of the PWHC, complementing domain-specific clinical outcomes by indicating whether postpartum health problems meaningfully limit a woman’s daily activities, participation, or quality of life [22].

### 4.2. Mental Health

Maternal mental health provides perhaps the clearest example of a postpartum condition that cannot be adequately addressed through a single early visit. Postpartum depression is common. Liu et al.’s systematic review and meta-analysis estimated a substantial global burden, although prevalence varies considerably by setting and measurement method [23]. Psychological distress can affect the woman directly and may also influence relationships, caregiving, breastfeeding experiences, and family functioning. Postpartum psychological morbidity also extends beyond depression and anxiety. For some women, childbirth itself may be experienced as traumatic, particularly following emergency obstetric intervention, severe maternal or neonatal complications, perceived loss of control, inadequate communication, or experiences of disrespectful care [25]. Birth-related post-traumatic stress symptoms and post-traumatic stress disorder (PTSD) may subsequently affect maternal wellbeing, relationships, infant care, future reproductive decisions, and engagement with healthcare [25,26]. Recognition of such experiences is important because psychological consequences may persist even when physical recovery appears clinically satisfactory. Postpartum mental-health pathways should therefore be sufficiently broad to recognize trauma-related symptoms as well as depression and anxiety, with appropriate assessment, referral, and follow-up when indicated. Mental-health care therefore needs to progress beyond screening coverage. Screening is valuable only when followed by clinical assessment, appropriate referral, treatment, and reassessment. A system that identifies depression but offers no accessible response merely documents unmet need. In this respect, postpartum mental-health care provides a clear example of the closed-loop principle developed later in the PWHC: identification should lead to assessment, assessment to an assigned care pathway, and referral to confirmation that the woman has entered appropriate treatment or follow-up. The relevant measure is therefore not only whether screening occurred, but whether a positive result resulted in subsequent assessment and clinically appropriate management [23,27]. The relationship between breastfeeding and mental health further demonstrates the interconnected nature of postpartum health domains. Yuen et al.’s systematic review showed that breastfeeding and maternal mental health are closely associated, although the relationship is complex and bidirectional [24]. Difficult breastfeeding experiences may worsen distress, while depression and anxiety may make breastfeeding more difficult. This reinforces the need to avoid evaluating maternal wellbeing solely through infant-feeding outcomes.

Cultural and interpersonal factors also influence mental-health care. Qualitative evidence from Mongolia provides an especially important example. Withers et al. reported that women experiencing postpartum depression wanted healthcare professionals to ask directly about their feelings and described barriers to recognition and help-seeking [27]. Their findings suggest that service availability alone is insufficient. Effective care requires professionals who recognize emotional distress and create socially acceptable opportunities for women to discuss it. Mental-health assessment within the PWHC should therefore be conceived as a pathway rather than an instrument: recognition should lead to assessment, assessment to appropriate intervention, and intervention to continued follow-up where necessary.

### 4.3. Pregnancy as a Window into Cardiometabolic Health

Pregnancy can function as an early physiological stress test that identifies women with increased susceptibility to later cardiovascular and metabolic disease. Adverse pregnancy outcomes, including hypertensive disorders of pregnancy and gestational diabetes mellitus (GDM), are associated with elevated future cardiometabolic risk and therefore provide an opportunity for earlier prevention [28,29]. Importantly, an elevated future risk should not be equated with established cardiovascular or metabolic disease. The implications for postpartum care depend on whether abnormalities persist after pregnancy, the woman’s broader risk profile, and the need for longer-term preventive surveillance.

Persistent hypertension after a hypertensive disorder of pregnancy provides one example of this distinction. Abnormal postpartum blood-pressure patterns have been documented following new-onset hypertensive disorders, supporting continued surveillance beyond hospital discharge [30]. Women with persistent hypertension require active clinical assessment and management, whereas those whose blood pressure normalizes may require less intensive but appropriately documented longer-term cardiovascular-risk assessment and preventive care. Similarly, GDM identifies an increased risk of subsequent dysglycemia and type 2 diabetes, but does not imply that all affected women have persistent diabetes after delivery. Postpartum metabolic assessment is therefore important for distinguishing women with ongoing abnormal glucose metabolism from those requiring longer-term preventive surveillance [31,32].

These conditions illustrate the “missing patient” phenomenon particularly clearly: pregnancy may identify a clinically important risk or abnormality, yet longitudinal responsibility for that finding may become ambiguous once maternity care ends. The PWHC therefore proposes risk-stratified continuation rather than uniform specialist follow-up. Depending on individual findings and local health-system organization, the appropriate destination may be routine primary care with documented surveillance, structured cardiovascular or metabolic prevention, or specialist care for women with persistent abnormalities, established disease, substantial comorbidity, or higher clinical risk. The central principle is not that every adverse pregnancy outcome requires the same intensity of care, but that clinically relevant pregnancy-associated risks should have an explicit and proportionate long-term care destination.

### 4.4. Sexual and Reproductive Health

Sexual and reproductive healthcare remains another essential component of the postpartum continuum. Contraception is particularly important, but reproductive care should not be reduced to contraception alone. A systematic review and meta-analysis by Dev et al. demonstrated that postpartum contraceptive use remains highly variable across low- and middle-income countries [33]. Implementation challenges also extend beyond availability. Nabhan et al.’s mixed-methods systematic review identified numerous barriers and facilitators influencing the scaling and sustainability of post-pregnancy family-planning programmes, reinforcing the importance of integration, counselling quality, provider capacity, and health-system organization [34]. These findings suggest that postpartum reproductive care should emphasize autonomy and continuity rather than simply uptake. Women need accurate information, meaningful choice of methods, opportunities to discuss reproductive intention, and access to care appropriate to their circumstances. Sexual health should also encompass dyspareunia, vaginal symptoms, pelvic-floor morbidity, changes in libido, and concerns about resuming sexual activity. Such topics may be neglected when the postpartum encounter is dominated by infant feeding, wound review, or contraception alone. The underlying principle is that childbirth does not end a woman’s sexual and reproductive healthcare needs; it changes their context.

### 4.5. Breastfeeding, Nutrition, and Maternal Wellbeing

Breastfeeding is a major component of postnatal care and has important health implications for both mother and infant. Yet postpartum systems should avoid reducing maternal success to breastfeeding performance. Women may experience nipple trauma, mastitis, pain, exhaustion, anxiety about milk supply, nutritional concerns, sleep disruption, and conflict between breastfeeding recommendations and employment demands. As noted above, breastfeeding experiences and mental health are closely intertwined [24]. A woman-centered approach should therefore support breastfeeding where desired and medically appropriate while recognizing maternal wellbeing as an outcome. Women who cannot breastfeed, encounter persistent difficulty, or choose alternative feeding approaches should not experience postpartum care as a system in which their health is secondary to feeding performance. Maternal nutrition should likewise be considered independently of breastfeeding status. The postpartum period may involve continuing nutritional needs associated with recovery from pregnancy and childbirth, including replenishment after blood loss or anemia, adequate energy and nutrient intake during physical recovery, and additional nutritional demands where breastfeeding is established. Nutritional care may also intersect with cardiometabolic health, particularly for women with pregnancy-associated metabolic risk. Importantly, nutritional adequacy is influenced not only by clinical advice but also by caregiving demands, household resources, food access, cultural practices, and return to employment. Postpartum nutritional assessment and support should therefore be individualized according to maternal health status, feeding practices, dietary circumstances, and social context [24]. This domain again illustrates why postpartum health is inherently multidimensional. A breastfeeding problem may simultaneously be a pain problem, a mental-health problem, an employment problem, and a social-support problem.

### 4.6. Social and Occupational Wellbeing

The postpartum period is not merely a biological recovery phase. It is also a major social transition. Women may experience changes in sleep, relationships, household workload, financial security, identity, and employment. Care responsibilities can directly influence whether women have time to attend appointments, undertake physical rehabilitation, prepare healthy food, sleep adequately, or seek mental-health treatment. The ILO’s global work on care policies demonstrates that access to maternity protection, parental leave, breastfeeding support, childcare, and decent working conditions remains uneven [16]. These policies are not traditionally categorized as postpartum healthcare, yet they influence the ability of women to follow clinical recommendations and recover adequately. The PWHC therefore includes social and occupational wellbeing not because healthcare professionals can solve every social problem, but because they should recognize when social conditions make recovery or continued treatment unrealistic. A clinician who identifies persistent hypertension but ignores that a woman cannot attend follow-up because of insecure employment has identified only part of the problem.

## 5. Translating the Continuum Across Asian Health Systems

The following country sections are intended as illustrative case examples rather than a comparative survey of Asian postpartum systems. The four countries differ substantially in institutional structure, resource availability, geography, and service delivery, and they do not capture the full diversity of the region. Their purpose is to demonstrate how selected continuity functions can be operationalized under different health-system conditions.

### 5.1. Japan: Institutionalizing Postpartum Support

Japan offers an important example of postpartum support becoming formally embedded in national and municipal policy. The Children and Families Agency has developed and updated national guidance for antenatal and postpartum support and postpartum-care services, with revised guidance issued in 2026 [35]. Japan’s approach includes structured postpartum support beyond the conventional outpatient consultation and allows multiple forms of service delivery. Suzuki has described the Japanese Postpartum Care Program and its development as a formal system supporting maternal physical recovery, mental wellbeing, infant-care adjustment, breastfeeding, and family transition [36]. Japan is therefore useful precisely because it demonstrates that postpartum care can be recognized as a distinct policy and service category rather than treated as an informal extension of obstetric care. However, institutionalization raises a further question. Formal postpartum support is predominantly designed around early recovery and adaptation. The longer-term transition into cardiovascular prevention, metabolic care, persistent mental-health treatment, pelvic-floor rehabilitation, and longitudinal reproductive care still requires linkage with other parts of the healthcare system. Japan therefore demonstrates that the ‘missing patient’ problem is not simply a product of insufficient resources. A well-resourced system can institutionalize postpartum care and still face the challenge of linking that care to lifelong women’s health.

### 5.2. Sri Lanka: Community Continuity

Sri Lanka offers a different model. Its maternal-health architecture relies strongly on community-level public-health services and the Public Health Midwife. The WHO has described Sri Lanka’s national maternal-health programme as extending from preconception through pregnancy, childbirth, the postpartum period, family planning, and well-woman services [37]. Importantly, continuity is supported through home visits and community-level contact rather than relying exclusively on women returning to hospital facilities. National survey analysis has documented high overall postnatal-care coverage in Sri Lanka while also showing that complete coverage remains imperfect [38]. Siriwardhana et al. further demonstrated socioeconomic and sector-related inequalities in postnatal home visits by Public Health Midwives, illustrating that even strong community systems can contain important access gaps [39]. Sri Lanka therefore contributes two lessons to the PWHC. First, continuity does not require hospital continuity. Community professionals can act as connective infrastructure between the household, primary care, and referral services. Second, a universal programme should not be assumed to produce universal access. Equity needs to be measured within the system rather than inferred from national coverage alone. The transferable lesson is not necessarily the precise Sri Lankan Public Health Midwife structure. It is the function of a geographically accountable health professional who actively maintains contact with women rather than waiting for them to re-enter care independently.

### 5.3. Thailand: Integrating Postpartum Health Within UHC

Thailand provides a third health-system lens. Its longstanding progress toward UHC and integration of sexual and reproductive healthcare offer a comparatively strong platform on which broader postpartum continuity could be built [40]. The relevant question in Thailand is therefore less about establishing access from the beginning and more about connecting services that already exist. Sothornwit et al. provide an instructive example. During the COVID-19 period, individualized postpartum scheduling combined with telemedicine increased postpartum contact and contraceptive uptake at a tertiary hospital in Thailand [41]. The study also found that improvement in some outcomes did not automatically translate into improvement across all maternal and infant outcomes, reinforcing the importance of evaluating postpartum interventions multidimensionally. Social support is another important component of the Thai context. Kim et al. found that both conventional and online social support were associated with stress and depression among postpartum women during the pandemic [42]. These findings highlight that postpartum wellbeing cannot be addressed by clinical appointments alone. Thailand therefore illustrates the integration challenge. UHC creates a platform, but maternity care, primary care, reproductive health, mental health, rehabilitation, digital services, and social support need to operate as parts of a pathway rather than as parallel systems.

### 5.4. Mongolia: Continuity Across Geographic Distance

Mongolia illustrates why a universal postpartum framework cannot mean identical service delivery. Its large territory and dispersed population make repeated access to centralized facilities unrealistic for many families. Telemedicine has therefore been used for maternal and newborn healthcare in remote provinces. Baatar et al. described an early telemedicine system connecting geographically distant providers with specialist expertise, demonstrating how technology can reduce spatial barriers within maternal and newborn services [43]. However, connectivity alone is not continuity. The qualitative findings of Withers et al. regarding postpartum depression demonstrate that women may remain underserved even when health services technically exist if symptoms are not recognized, women do not feel able to seek help, or providers do not initiate appropriate conversations [27]. Mongolia therefore shows that digital and remote healthcare must be combined with trusted local professionals, clear referral pathways, and culturally appropriate communication. The central lesson is that health systems should adapt continuity to geography rather than requiring geography to adapt to conventional service models.

### 5.5. From Country Models to Common Health-System Functions

The four exemplars illustrate different responses to the shared problem of postpartum discontinuity, but they should not be interpreted as representing the range or distribution of postpartum-care models across Asia. Japan demonstrates institutionalization, Sri Lanka demonstrates community continuity, Thailand demonstrates integration within UHC, and Mongolia demonstrates adaptation to geographic constraint. None should be treated as the single model for Asia. The PWHC therefore proposes common functions rather than common organizational forms. Every system needs a way to identify postpartum morbidity, maintain appropriate contact, determine when problems require escalation, and ensure that unresolved or long-term risks are transferred to a continuing source of care. The professional performing these functions may differ between systems. It may be a midwife, public-health nurse, family physician, obstetric team, community health worker, digital service, or multidisciplinary combination. What matters is not whether the service architecture looks identical. What matters is whether women with ongoing needs remain connected to care.

## 6. The Postpartum Women’s Health Continuum

The PWHC was developed by integrating two levels of the preceding narrative synthesis. First, recurrent postpartum needs identified across the literature were grouped into six health domains: physical recovery and function, mental health, cardiometabolic health, sexual and reproductive health, breastfeeding and nutrition, and social and occupational wellbeing. Second, evidence concerning service delivery, care transitions, referral, and variation in postpartum risk was synthesized to identify the health-system functions required to maintain continuity across these domains. This process informed three organizing principles: universal early continuity, risk-stratified follow-up, and explicit handover. The framework was informed by existing postpartum-care guidance but was not derived from a single pre-existing model, and its final structure was refined through author discussion and comparison across the four health-system exemplars. Universal early continuity means that all women should leave maternity care with information about expected recovery, warning symptoms, early follow-up arrangements, and a clearly identifiable route back into professional care. This principle is consistent with WHO recommendations emphasizing multiple postnatal contacts and a positive postnatal experience [3,4]. Risk-stratified follow-up means that women should not all be required to follow an identical year-long schedule. Systematic review evidence suggests that different postpartum delivery strategies can be effective under different circumstances [8], while women’s engagement with postnatal care varies according to both need and context [12]. Women with uncomplicated recovery may need relatively little additional contact after routine early postnatal care, whereas women with hypertensive disorders, GDM, significant hemorrhage, persistent pelvic-floor morbidity, positive mental-health screening, breastfeeding complications, chronic disease, social vulnerability, or other identified risks may require substantially longer follow-up.

The third principle, explicit handover, is the most important. Postpartum care should end with a destination rather than simply with the expiration of an obstetric timetable. For a woman whose recovery is complete, that destination may be routine primary or women’s healthcare. For persistent depression, it should be an active mental-health pathway. For GDM, it should include a documented plan for metabolic surveillance. For women with a history of GDM, postpartum care should include not only appropriate metabolic assessment and longer-term surveillance, but also individualized support for modifiable lifestyle factors, including healthy dietary patterns, physical activity, and weight management where appropriate, with the intensity of follow-up determined by postpartum findings and overall metabolic risk. For hypertensive pregnancy, it should include blood-pressure and cardiovascular-risk follow-up. For persistent pelvic-floor symptoms, it may involve physiotherapy, gynecology, urogynecology, or another appropriate service. The distinction between a referral being offered and a transition being completed is fundamental. A successful handover should therefore involve closed-loop transfer of responsibility, such that the referring service can confirm that the receiving provider or service has accepted responsibility and that the woman has entered the intended pathway of care. Depending on the health-system context, evidence of a completed transition may include documented referral acceptance, a scheduled or completed first appointment, confirmation that follow-up occurred, or documentation that ongoing management has been assumed by the receiving service. Transition programmes and active primary-care scheduling demonstrate that health systems can increase the probability that women actually enter continuing care rather than merely receiving advice to do so [9,10]. Similarly, evidence from women following high-risk pregnancies shows why patients should not be expected to coordinate fragmented services independently [11]. The PWHC therefore conceptualizes continuity as a health-system responsibility.

**Figure 1:**
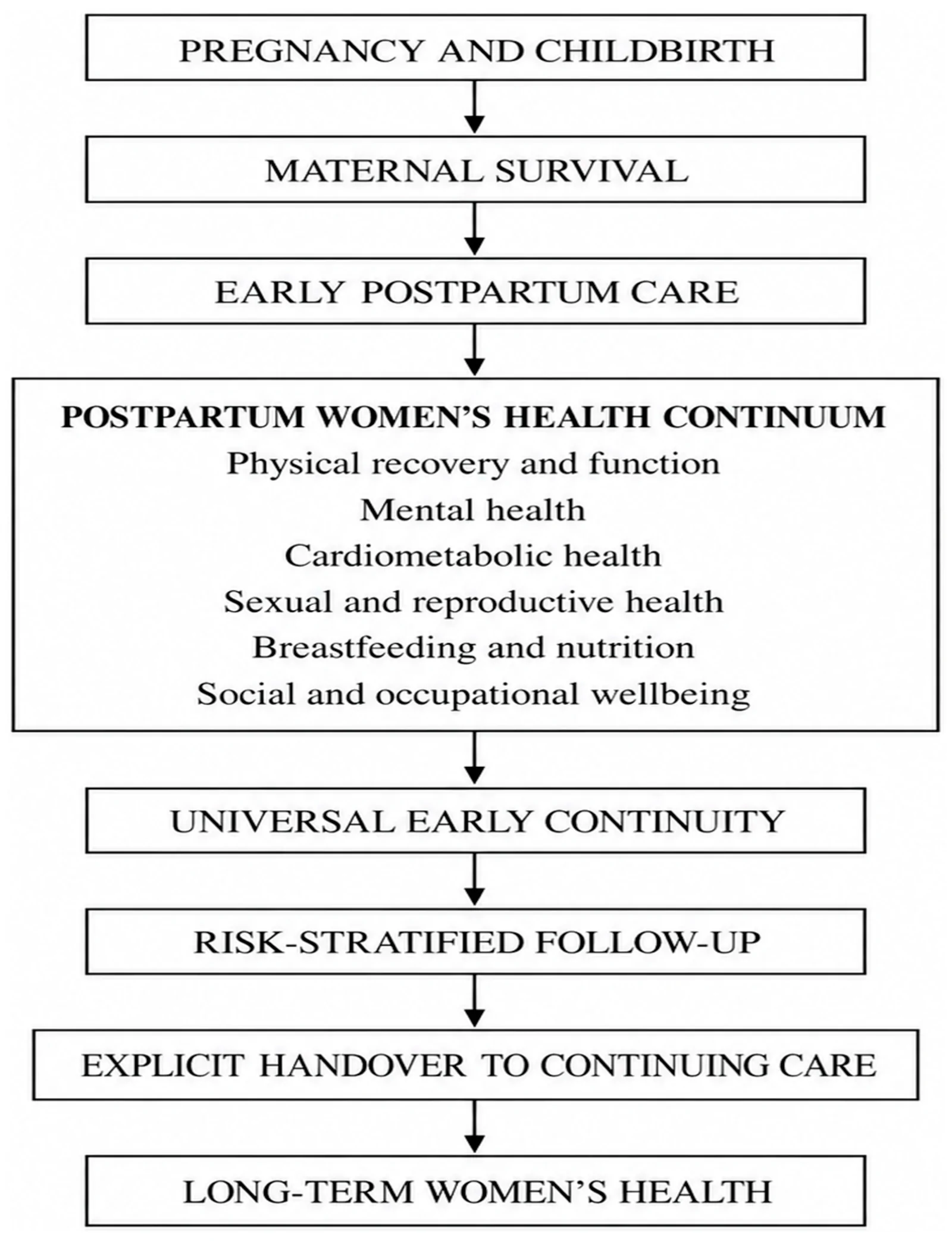
The Postpartum Women’s Health Continuum.

Figure 1 conceptualizes childbirth as a transition rather than an endpoint of women’s healthcare. After early postpartum care, women may require continued attention across six interconnected domains: physical recovery and function, mental health, cardiometabolic health, sexual and reproductive health, breastfeeding and nutrition, and social and occupational wellbeing. The continuum is supported by universal early continuity, risk-stratified follow-up, and explicit handover of unresolved needs into continuing care, with the goal of improving long-term women’s health.

### 6.1. Timing as Transition Checkpoints Rather than Mandatory Visits

The PWHC does not propose a new universal schedule of mandatory face-to-face postpartum appointments. Existing WHO recommendations provide an evidence-based foundation for the immediate and early postnatal period, emphasizing multiple contacts, assessment of maternal and newborn wellbeing, and continuity of care during the weeks following childbirth [3,4]. Comparative review of international postnatal-care guidelines likewise demonstrates considerable variation in the recommended timing, number, and content of postpartum contacts across health systems [5]. Evidence from systematic reviews further suggests that different postpartum-care delivery strategies, including earlier follow-up, home-based care, multidisciplinary approaches, and telehealth-supported models, may be appropriate under different clinical and organizational circumstances rather than requiring a single standardized schedule for all women [8].

In contrast, the later time points discussed within the PWHC—approximately three months, six months, and one year postpartum—are proposed by the authors as illustrative transition checkpoints rather than as established guideline recommendations. Their purpose is to provide pragmatic opportunities for health systems to determine whether unresolved maternal-health needs have improved, entered an appropriate management pathway, or been successfully transferred into continuing care. The need for such follow-up is supported by evidence showing that postpartum engagement is influenced by clinical need, social circumstances, access barriers, and health-system organization [12], and that women may experience fragmented transitions between maternity care, primary care, and specialist services after childbirth [9,10,11]. The precise timing and mode of these checkpoints should therefore be adapted to individual risk, local service structures, and existing care pathways.

An illustrative transition checkpoint at approximately three months may be useful for reassessing unresolved physical or psychological symptoms, confirming follow-up after gestational diabetes mellitus or hypertensive disorders of pregnancy, and reviewing functional recovery. This is particularly relevant because pregnancy-associated hypertensive and metabolic conditions may confer continuing cardiovascular and metabolic risk after delivery and require surveillance beyond the conclusion of routine maternity care [28,29,30,31,32]. A later checkpoint around six months may be appropriate for women with persistent morbidity, ongoing breastfeeding-related difficulties, substantial care burden, or challenges associated with return to work, recognizing that postpartum recovery can extend well beyond the early postnatal period and may involve interacting physical, psychological, nutritional, and social needs [17,20,21,23,24].

By approximately one year postpartum, women with continuing pregnancy-associated or other unresolved health risks should ideally have an established long-term care destination, whether within primary care, mental-health services, rehabilitation, reproductive healthcare, cardiovascular or metabolic prevention, or another appropriate service. Evidence from postpartum transition programmes and active primary-care scheduling indicates that health-system design can improve the likelihood that women actually enter continuing care rather than merely receiving advice or referral [9,10]. These transition functions do not necessarily require additional standalone postpartum visits; they may be incorporated into primary-care consultations, reproductive-health services, infant-health contacts, vaccination encounters, community-based care, digital follow-up, or chronic-disease programmes. The objective is therefore not to prescribe more healthcare encounters for every woman, but to reduce the number of women with clinically meaningful needs who are lost between services.

### 6.2. Nurses and Midwives as Continuity Infrastructure

A comprehensive postpartum model would be unsustainable if interpreted as a demand for repeated specialist obstetric review. Nurses and midwives are therefore central to the PWHC. The WHO’s recent implementation guidance on midwifery models of care emphasizes continuity, person-centered relationships, and appropriate professional autonomy across reproductive health services [15]. These principles are highly compatible with postpartum continuity. Within the local scope of practice and according to professional training and regulatory context, appropriately trained nursing and midwifery professionals can assess physical recovery, measure blood pressure, support breastfeeding, identify mental-health concerns, discuss contraception within local scope of practice, assess social circumstances, provide health education, and determine when medical escalation is needed. The strength of nursing and midwifery is not merely that these professionals may provide lower-cost care than specialist physicians. Their roles can connect domains traditionally separated by medical specialties. A breastfeeding consultation may reveal depression, pain, exhaustion, family conflict, or anxiety. A home visit may reveal mobility barriers, inadequate support, or environmental conditions invisible during a clinic appointment. Sri Lanka’s Public Health Midwife system illustrates the potential of community-level continuity [37,38,39]. Japan’s postpartum-care programmes similarly demonstrate how postpartum support can involve multidisciplinary arrangements rather than obstetricians alone [34,35,36]. The appropriate model is therefore not professional substitution but task differentiation, coordination, and escalation within locally defined scopes of practice.

### 6.3. Digital Health as an Enabler, Not a Replacement

The postpartum period is particularly suitable for selective digitalization because attending healthcare while caring for a newborn carries substantial opportunity costs. Telemedicine, secure messaging, remote blood-pressure monitoring, digital mental-health screening, appointment reminders, health education, and referral navigation may reduce unnecessary travel and improve access. Thailand demonstrates that telemedicine-supported individualized scheduling can increase postpartum engagement [41]. Mongolia demonstrates the value of telemedicine where geography constrains access to specialist care [43]. However, digital care also carries risks. Women without reliable internet access, suitable devices, digital literacy, private space, or confidence using digital health tools may be disadvantaged if remote care replaces rather than supplements physical services. Some postpartum problems also require direct examination, laboratory testing, observation of breastfeeding, rehabilitation assessment, or therapeutic relationships that cannot be reproduced fully through digital communication. The PWHC therefore supports a digital-plus-human model in which technology reduces friction but does not transfer responsibility back to the woman. Digital systems should make it easier for a patient to reach appropriate human care, not merely make it easier for health systems to send information.

### 6.4. Measuring the Missing Patient

The broader postpartum agenda also requires better measurement. Maternal mortality is a powerful policy indicator partly because it is conceptually clear and internationally comparable. Recovery, functioning, psychological wellbeing, reproductive autonomy, cardiometabolic prevention, and social participation are more difficult to summarize within a single metric. Difficulty of measurement, however, should not determine clinical importance. The future postpartum measurement agenda should therefore complement mortality and early postnatal coverage with indicators reflecting whether identified maternal-health needs are recognized, acted upon, and successfully managed. Within the PWHC, measurement can be considered at both the process and outcome levels. Illustrative process indicators could include the proportion of women with hypertensive disorders of pregnancy who complete recommended blood-pressure or cardiovascular-risk follow-up; the proportion of women with gestational diabetes mellitus who complete indicated postpartum metabolic assessment; the proportion of women with a positive mental-health screen who receive subsequent clinical assessment or treatment; and the proportion of referrals requiring continuing care that are successfully closed through documented acceptance, first contact, or confirmation that the woman entered the intended care pathway [9,10,23,28,29,30,31,32]. These measures would evaluate whether the health system actually performed the continuity functions proposed in the PWHC rather than simply whether screening or referral occurred. Outcome indicators could complement these process measures by assessing whether clinically meaningful problems persisted, improved, or were resolved. Examples include persistence or improvement of functionally important pelvic-floor symptoms, urinary incontinence, pain, or other limitations in physical recovery [17,20,21]; persistence or improvement of clinically important mental-health symptoms [23,27]; and unresolved sexual or reproductive-health needs despite contact with services [33,34]. Such indicators may be particularly informative when expressed in terms of functional consequence and patient-reported wellbeing rather than service utilization alone. To translate the PWHC into a more operational and evaluable framework, a small set of illustrative process and outcome indicators is proposed in Table 1. These indicators are not intended to constitute a validated or universally standardized measurement set. Rather, they demonstrate how the major components of the PWHC could be assessed in practice, including whether identified maternal-health needs lead to appropriate follow-up, successful transfer into continuing care, and improvement or persistence of clinically meaningful morbidity. The specific definitions, denominators, timing, and data sources would need to be adapted to local health-system structures and evaluated in future implementation research.

Closed-loop referral provides an operational expression of the PWHC principle of explicit handover. If maternity care identifies a woman requiring continuing management, the relevant question is not only whether a referral was issued, but whether the intended transition was completed. A closed loop may therefore be documented through evidence that the receiving service accepted the referral, that an initial follow-up contact was scheduled or completed, or that responsibility for continuing management was formally assumed. The specific indicator may vary according to local health-system architecture, but the central requirement is confirmation that the woman entered the intended care pathway. This distinction allows explicit handover to be measured as a process of continuity rather than inferred from referral activity alone. A screening programme may report excellent coverage while producing little benefit if positive results are not acted upon. A referral system may generate large numbers of referrals without knowing whether care occurred. A postpartum visit may be completed without addressing the health problem most important to the woman. The more meaningful question is whether an identified need was resolved, managed, or successfully transferred.

### 6.5. Avoiding Overmedicalization, Over-Standardization, and Workforce Burden

Expanding the concept of postpartum health creates legitimate risks. The first is overmedicalization. New motherhood often includes fatigue, uncertainty, relationship adjustment, sleep disruption, and emotional change. Not every discomfort should be transformed into pathology. The purpose of the PWHC is not to create an unrealistic expectation of symptom-free postpartum life, but to identify problems that are persistent, severe, functionally important, or responsive to intervention. The second risk is over-standardization. Asia contains enormous cultural, demographic, geographic, and socioeconomic diversity. Even within Japan, Sri Lanka, Thailand, and Mongolia, substantial differences exist between urban and rural communities, social groups, ethnic populations, and generations. Cultural practices concerning postpartum rest, family involvement, breastfeeding, diet, mental health, and help-seeking should not be reduced to national stereotypes. The third risk is workforce overload and implementation without sufficient downstream capacity. Adding additional screening requirements without referral capacity can create administrative work without improving outcomes. Every assessment should therefore have an anticipated response. If a woman screens positive for depression, the system needs a treatment pathway. If persistent incontinence is identified, referral or rehabilitation must be accessible. If blood pressure remains elevated, there must be clear medical responsibility. The feasibility of implementing the PWHC therefore depends not only on the capacity to identify postpartum needs, but also on the capacity of the health system to respond to them. The fourth risk is shifting responsibility onto women. Educational materials, apps, self-monitoring, and reminders can be useful, but they can also subtly redefine continuity as the woman’s responsibility to navigate a fragmented system. The PWHC takes the opposite position. Continuity should be understood as a property of the healthcare system, not as evidence that a postpartum woman successfully navigated it.

### 6.6. Implications for Research and Policy

The proposed framework has several implications for future maternal-health research. Longitudinal studies should examine postpartum morbidity beyond the conventional early postnatal period, particularly pelvic-floor health, mental health, cardiometabolic risk, functional recovery, sexual health, and patient-reported wellbeing. Evidence that pelvic-floor dysfunction can remain clinically important many months postpartum [17,20,21] demonstrates the limitations of very short follow-up windows. Implementation studies should also evaluate which combinations of maternity care, primary care, nursing, midwifery, community services, and digital health provide effective continuity at reasonable cost. Existing evidence on postpartum delivery strategies, primary-care transitions, scheduling, and high-risk pregnancy follow-up provides an important foundation for such work [8,9,10,11,31]. Cardiometabolic conditions offer especially strong opportunities for intervention research. Pregnancy already identifies women with hypertensive disorders or GDM, and the association of adverse pregnancy outcomes with later cardiovascular risk is increasingly well established [28,29,30,31,32]. The unanswered question is how to convert this knowledge into durable care pathways. Mental-health programmes should similarly move beyond screening implementation to examine treatment completion and longer-term outcomes [23,27]. Research on postpartum family planning should focus not only on method uptake but also on integration, autonomy, continuity, and implementation conditions [33,34]. Economic evaluation will also be necessary. The PWHC may require additional resources, but many functions could potentially be integrated into existing primary-care, child-health, community, or digital encounters rather than creating an entirely new layer of services. Moreover, postpartum policy should increasingly engage sectors beyond health ministries. Employment, maternity and parental leave, childcare, social protection, transportation, digital infrastructure, and gender policy all influence whether women are capable of acting on medical advice [16]. Postpartum women’s health is therefore not simply an obstetric programme to be enlarged. It is a test of whether health and social systems can maintain continuity across one of the most significant transitions in a woman’s life.

The PWHC should be regarded as a proposed conceptual framework rather than an established or validated model of care. It has yet to be prospectively implemented or evaluated for effectiveness, and the present review therefore cannot determine whether adoption of the framework would improve continuity, maternal morbidity, patient-reported outcomes, or health-system efficiency. Future research should evaluate the framework in stages. Initial studies could assess feasibility, acceptability, and contextual fit among postpartum women, nurses, midwives, obstetricians, primary-care professionals, and health-system decision-makers. Subsequent implementation studies could examine how the framework can be integrated into existing maternity, primary-care, community, and digital-care pathways, including the resources and workforce implications of doing so. Prospective observational or intervention studies could then assess whether implementation improves measurable continuity processes, such as completion of risk-stratified follow-up, treatment after positive mental-health screening, and successful closed-loop referral, and whether these improvements are associated with better maternal physical, psychological, reproductive, cardiometabolic, and functional outcomes. Evaluation across contrasting health-system settings will also be important to determine which components of the PWHC are transferable and which require local adaptation.

## 7. Conclusions

Global maternal health has rightly spent decades asking how to prevent women from dying during pregnancy and childbirth, and this goal remains urgent and unfinished. Yet the next challenge is to ensure that survival is followed by recovery, wellbeing, and continuity of care beyond the conventional boundaries of maternity services. The experiences of Japan, Sri Lanka, Thailand, and Mongolia illustrate that postpartum continuity can be supported through different health-system architectures, including formalized postpartum programmes, community-based follow-up, integration within universal health coverage, and models adapted to geographic distance. These examples are illustrative rather than representative of postpartum care across Asia. The proposed PWHC brings these observations together into a conceptual health-system framework centered on universal early continuity, risk-stratified follow-up, nurse- and midwife-enabled coordination within local scopes of practice, appropriate use of digital health, and explicit handover of unresolved maternal-health needs into continuing care. The framework is intended to support future thinking about how maternity care can connect more reliably with primary, specialist, community, and other longitudinal services; it has yet to be prospectively implemented or validated as an effective model of care. Future research should therefore assess the feasibility, acceptability, adaptability, and effectiveness of the PWHC across different health-system settings, including whether its proposed continuity processes and indicators are associated with improved maternal outcomes. As 2030 approaches, maternal-health policy should continue to build upon the goal of survival while ensuring that the physical, psychological, reproductive, cardiometabolic, functional, and social consequences of pregnancy remain visible after maternity care ends. The postpartum woman should not become the “missing patient” once the newborn becomes visible; childbirth should mark a transition in women’s healthcare, not its endpoint.

## Figures and Tables

**Table 1:** Illustrative indicators for evaluating the Postpartum Women’s Health Continuum.

PWHC Component	Illustrative Indicator	Type
Universal early continuity	Proportion of women discharged with documented follow-up arrangements and an identifiable route back to care	Process
Risk-stratified follow-up	Proportion of women with hypertensive disorders of pregnancy completing indicated postpartum blood-pressure or cardiovascular-risk follow-up	Process
Risk-stratified follow-up	Proportion of women with gestational diabetes mellitus completing indicated postpartum metabolic assessment	Process
Mental-health continuity	Proportion of women with a positive mental-health screen receiving subsequent clinical assessment and, where indicated, treatment	Process
Explicit handover	Proportion of referrals for continuing maternal care completed through closed-loop confirmation	Process
Physical recovery and function	Proportion of women with postpartum health problems reporting persistent, improved, or resolved limitations in daily, social, caregiving, or occupational functioning	Outcome
Sexual and reproductive health	Proportion of women with unresolved sexual or reproductive-health needs after postpartum contact	Outcome
Long-term continuity	Proportion of women with pregnancy-associated long-term risk who have an identified continuing-care provider or service	Process

## Data Availability

No new data were created or analyzed in this study. Data sharing is not applicable to this article.

## Abbreviations

The following abbreviations are used in this manuscript:

ACOG	American College of Obstetricians and Gynecologists
GDM	Gestational diabetes mellitus
ILO	International Labor Organization
NCD	Noncommunicable disease
PNC	Postnatal care
PTSD	Post-traumatic stress disorder
PWHC	Postpartum Women’s Health Continuum
SDG	Sustainable Development Goal
SRH	Sexual and reproductive health
UHC	Universal health coverage
WASH	Water, sanitation, and hygiene
WHO	World Health Organization
